# Dietary Patterns Are Associated with Cardiovascular and Cancer Mortality among Swiss Adults in a Census-Linked Cohort

**DOI:** 10.3390/nu10030313

**Published:** 2018-03-07

**Authors:** Jean-Philippe Krieger, Sophie Cabaset, Giulia Pestoni, Sabine Rohrmann, David Faeh, Swiss National Cohort Study Group

**Affiliations:** 1 Division of Chronic Disease Epidemiology, Epidemiology, Biostatistics and Prevention Institute, University of Zurich, Hirschengraben 84, CH-8001 Zurich, Switzerland; sophie.cabaset@uzh.ch (S.C.); giulia.pestoni@uzh.ch (G.P.); sabine.rohrmann@uzh.ch (S.R.); david.faeh@uzh.ch (D.F.); 2 Health Division, Nutrition and Dietetics, Bern University of Applied Sciences, Falkenplatz 24, CH-3012 Bern, Switzerland

**Keywords:** dietary patterns, dietary variety, mortality, dietary guidelines, public health

## Abstract

Defining dietary guidelines requires a quantitative assessment of the influence of diet on the development of diseases. The aim of the study was to investigate how dietary patterns were associated with mortality in a general population sample of Switzerland. We included 15,936 participants from two population-based studies (National Research Program 1A (NRP1A) and Monitoring of Trends and Determinants in Cardiovascular Disease (MONICA)—1977 to 1993) who fully answered a simplified 24-h dietary recall. Mortality data were available through anonymous record linkage with the Swiss National Cohort (follow-up of up to 37.9 years). Multiple correspondence analysis and hierarchical clustering were used to define data-driven qualitative dietary patterns. Mortality hazard ratios were calculated for all-cause, cancer and cardiovascular mortality using Cox regression. Two patterns were characterized by a low dietary variety (“Sausage and Vegetables”, “Meat and Salad”), two by a higher variety (“Traditional”, “High-fiber foods”) and one by a high fish intake (“Fish”). Males with unhealthy lifestyle (smokers, low physical activity and high alcohol intake) were overrepresented in the low-variety patterns and underrepresented in the high-variety and “Fish” patterns. In multivariable-adjusted models, the “Fish” (hazard ratio = 0.82, 95% CI (0.68–0.99)) and “High-fiber foods” (0.85 (0.72–1.00)) patterns were associated with lower cancer mortality. In men, the “Fish” (0.73 (0.55–0.97)) and “Traditional” (0.76 (0.59–0.98)) patterns were associated with lower cardiovascular mortality. In summary, our results support the notion that dietary patterns affect mortality and that these patterns strongly cluster with other health determinants.

## 1. Introduction

With the worldwide rise in non-communicable diseases, nutritional epidemiology has gained increasing importance. While classic approaches focusing on individual nutrients, foods or food groups are useful, they cannot account for the joint effects of the multiple components of diets. Therefore, the study of dietary patterns—i.e., the description of diets according to the combination of foods consumed—has become common practice [1]. Simple scores have been developed to measure how diets fit predefined hypotheses (i.e., a priori methods, such as the Mediterranean Diet Score [2]). These scores, however, do not identify diets specific to the population of interest [3]. In contrast, data-driven approaches (also called a posteriori approaches) generate hypothesis-free dietary patterns that better reflect a population’s diet [4].

Investigating the effect of diets on the development of non-communicable disease is a complex task that requires both quality dietary information and the long-term follow-up of individuals [5,6,7]. Switzerland offers an ideal setting for such investigation. First, health surveys have been regularly conducted in a representative sample of the Swiss population and have included comparable nutrition-related questions [8,9]. Moreover, the Swiss National Cohort (SNC), a national longitudinal research platform, links census records with federal death and migration records covering all residents of Switzerland, enabling the long-term follow-up of participants to health surveys [10]. Finally, the cultural diversity of Switzerland may impact dietary choices [3,11] and, thus, be an important source of variability of the dietary patterns in comparison to more homogenous populations. Therefore, although the association of dietary patterns with mortality outcomes has been widely investigated in other populations, our study setting may provide novel valuable information.

Our primary aim was to identify data-driven dietary patterns using 24-h recall data from two population-based national surveys. Based on this, we investigated whether the consumption of a dietary pattern was associated with demographic (sex, age, nationality), socioeconomic (education) and lifestyle (physical activity, alcohol consumption and smoking habits) factors. Finally, we leveraged the possibility of a long-term mortality follow-up (up to 37.9 years) to study the association between dietary patterns and all-cause, cancer or cardiovascular mortality. Together, our data offer valuable insights on the influence of dietary choices on long-term all-cause and disease-specific mortality.

## 2. Materials and Methods

### 2.1. Study Design and Participants

The overall study design consists of 2 population-based studies, National Research Program 1A (NRP1A) and Monitoring of Trends and Determinants in Cardiovascular Disease (MONICA), linked with the SNC. NRP1A is a community-based primary study on prevention of cardiovascular disease conducted from 1977 to 1979 [9]. Three waves of MONICA—an international multicenter study initiated by the World Health Organization—were conducted in Switzerland in 1984–1986 (MONICA1), 1988–1989 (MONICA2), and 1992–1993 (MONICA3) [8]. All studies comprised a self-administered simplified 24-hour recall checklist (see Section 2.2). Vital status and cause of death of the NRP1A and MONICA participants were followed through the SNC, a national longitudinal research platform linking census records with federal death and migration records covering all residents of Switzerland [10]. A procedure to match participants of NRP1A and MONICA with the SNC was described elsewhere [10]. The linkage is characterized by a high success rate, 93.8% and 97%, respectively [12,13]. Only participants who fully answered the 24-h recall checklist were included in the analysis (total: 15,936 out of 17,861; NRP1A: 6717 out of 8008; MONICA1: 3098 out of 3324; MONICA2: 3246 out of 3404; MONICA3: 2875 out of 3125). SNC and its linkage with MONICA and NRP1A were approved by the Ethics Committee of the Canton of Zurich (KEK-StV no. 13/06 and amendment of 12 June 2008).

### 2.2. Dietary Assessment

Diet was assessed once at study entry: participants were invited to attend a health examination in which they answered a self-administered questionnaire. The diet checklist comprised yes/no questions about food and beverage intake on the previous day. We considered the 12 following food groups, because they were consistently assessed across surveys: meat (white or red), sausage, fish, salad, vegetables, fruit, wholegrain products in the form of dark bread, chocolate, eggs, cheese, milk (as a drink), and yogurt (including other dairy products).

### 2.3. Identification of Dietary Patterns

We used a two-step procedure to identify dietary patterns in our sample. First, we applied Multiple Correspondence Analysis (MCA, a principal component method [14,15,16]) to the 12 nutrition questions. Breaks in the scree plot (Figure S1A) and a cumulative inertia above 40% (Figure S1B) were used as criteria to choose the number of principal components. Then, the 4 first principal components were used as input to hierarchical clustering using the Ward’s criterion [17]. While the number of clusters to retain is a debated topic [18], we used the decrease in within-inertia from *n* to *n* + 1 cluster (Figure S2A,B), and the interpretability of the partition, to choose 5 clusters. The defined partition was further consolidated using a k-means algorithm. Robustness of the 5 clusters was tested by randomly splitting the population in halves (data not shown). Dietary characteristics of each cluster are referred to as “dietary patterns”. Due to the nature of the dietary assessment as binary intakes, dietary patterns are qualitative and do not directly reflect the amount of foods consumed. Dietary variety was adapted from Kennedy et al. [19] and is the number of food groups reported by one participant divided by the total number of food groups in the dietary checklist (12 food groups). Dietary variety was averaged among all participants following each dietary pattern and expressed as percentage.

### 2.4. Association between Dietary Patterns and Demographics and Lifestyle Data

The following demographic and lifestyle data were assessed by questionnaires and were available across studies: age, sex, nationality (Swiss vs. Foreign), education (mandatory vs. upper secondary vs. tertiary), BMI (measured body mass index; <25 kg/m^2^ vs. 25 to 30 kg/m^2^ vs. ≥30 kg/m^2^), alcohol consumption (no; moderate (MONICA: drinking either wine or beer or cider; NRP1A: women drinking less than 20 g/day, men drinking less than 40 g/day); high (MONICA: drinking spirits or more than one sort of alcohol on the previous day; NRP1A: women drinking more than 20 g/day, men drinking more than 40 g/day)), physical activity (less than once a week vs. once a week vs. more than once a week), smoking status (never vs. former vs. light (<20 cigarettes/day) vs. heavy smoker (≥20 cigarettes/day)). Associations between these variables and the dietary patterns were investigated by testing whether the distribution (for continuous variables) or the proportion of categories (for qualitative variables) differ from those obtained from the overall population, using v-test as previously described [20].

### 2.5. Association between Dietary Patterns and Mortality

Cox proportional hazards regression models were fitted to assess the association between dietary patterns and all-cause, cancer, cardiovascular diseases or other-cause mortality. All Cox regression models were stratified by age to prevent violation of the proportional hazards assumption and by sex. Cox regression models were adjusted for potential confounders in a sequential manner; basic model: study (MONICA wave 1–3; NRP1A); multivariable model: study, BMI, nationality, education, smoking status, alcohol consumption and physical activity. None of the models violated the proportional hazards assumption on the basis of Schoenfeld residuals. In the case of missing information, an additional category was created for confounding variables with missing values. We performed sensitivity analysis by comparing the results of the Cox regression whether individuals who died within the first 5 years after study entry were included or excluded from the analysis. Finally, we estimated population-attributable fractions (PAFs) and rate advancement periods (RAPs) associated with dietary patterns for all-cause and specific mortality. On the basis of the Cox regression results, the population was split between the individuals consuming one of the three diets associated with reduced mortality (“Fish”, “Traditional”, “High-fiber foods”) and one of the other diets (“Sausage and Vegetables”, “Meat and Salad”). PAFs, RAPs and the corresponding confidence intervals were calculated as described elsewhere [21]. Because RAPs necessitate the age coefficient of the Cox regression, RAPs were calculated with non-age-stratified regression results. All descriptive and statistical analyses were conducted with R (R Core Team, Vienna, Austria) using R studio (v. 1.0.153, RStudio Team, Boston, MA, USA). MCA and hierarchical clustering used the FactoMineR package [22].

## 3. Results

### 3.1. Population Description

The study included 15,936 participants with a mean follow-up time of 25.5 years (Table 1). A majority of the participants were Swiss nationals (81.3%), received at least upper secondary education (65.4%) and were neither overweight nor obese (55.5%). Alcohol consumption and tobacco use were higher in males than females (alcohol: 71.4% in males, 39.2% in females; smoking: 42.6% in males, 29% in females). 

### 3.2. Identification of Dietary Patterns

**Figure 1:**
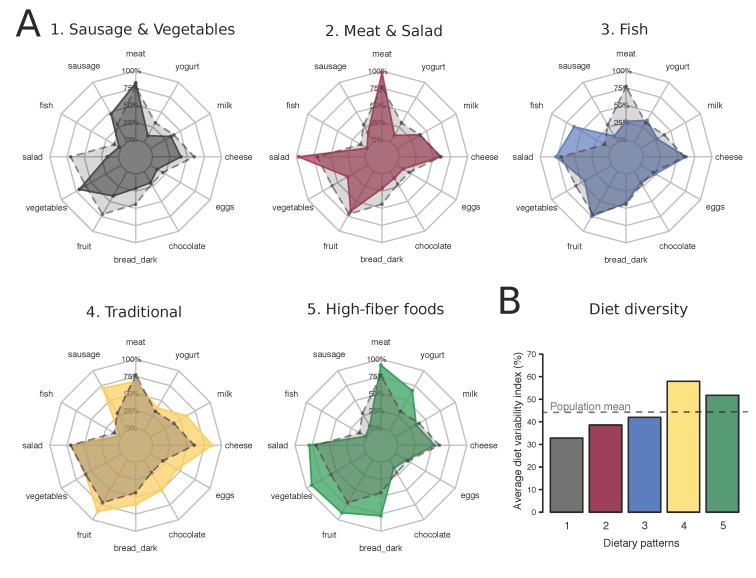
Dietary patterns of the Swiss Population (1977–1993; *N* = 15,936). (**A**) Percentage of individuals assigned to a dietary pattern who reported the consumption of the food groups. Grey dashed lines represent the percentage of individuals consuming the 12 food groups in the total study sample. Order of the dietary patterns is random and names were given based on food group consumption; (**B**) Average dietary variety (%) in individuals following each dietary pattern. Dietary variety is calculated for each participant as the number of food groups consumed divided by the total number of food groups in the diet checklist (12 food groups). Horizontal dashed line indicates the average dietary variety of the total study population.

In the simplified 24-hour recall checklist, meat (77.1%) was the most and fish (10.9%) the least frequently reported food item (Table S1). More than half of the participants reported consumption of fruits, vegetables, salad and cheese. MCA was used to explore the structure of the participants’ diets and the first four dimensions were considered for further analysis (Figure S1A–D). The two first dimensions of the MCA separated individuals by the overall variety of their diet, as evidenced by the factor loadings for the different food groups (Table S2) and the coordinates of the “yes” or “no” categories (Figure S1E). In addition, MCA separated individuals by the consumption of meat vs. fish (dimension 2), the consumption of energy-dense food groups vs. high-fiber food groups (dimension 3), and the consumption of salad (raw vegetables) vs. vegetables (cooked vegetables; Table S2 and Figure S1E,F). Subsequent hierarchical clustering allowed for defining five discrete dietary patterns (Figure S2A–D), described in Figure 1. Two dietary patterns (“Sausage and Vegetables” and “Meat and Salad”) were characterized by an overall low dietary variety (Figure 1B). In addition, in these two patterns, individuals eating sausages and cooked vegetables (“Sausage and Vegetables”) or meat and salad (“Meat and Salad”) were overrepresented (Figure 1A). A third dietary pattern (“Fish”) approached the average pattern of the population but was distinctly characterized by the intake of fish and the absence of meat-based products (Figure 1A). Finally, the two last dietary patterns were characterized by a high dietary variety (Figure 1B), but with an emphasis on different food groups: dairy products, eggs, chocolate and dark bread, sausages in the “Traditional” pattern and yogurt, salad, vegetables, fruits, and dark bread in the “High-fiber foods” pattern (Figure 1A). Each cluster grouped between 15.6% (cluster “Fish”) and 25.7% (cluster “Meat and Salad”) of the individuals included in the sample, indicating that all five dietary patterns were widespread in the studied population (Figure S2E).

### 3.3. Characterization of the Population Associated to Each Dietary Pattern

**Figure 2:**
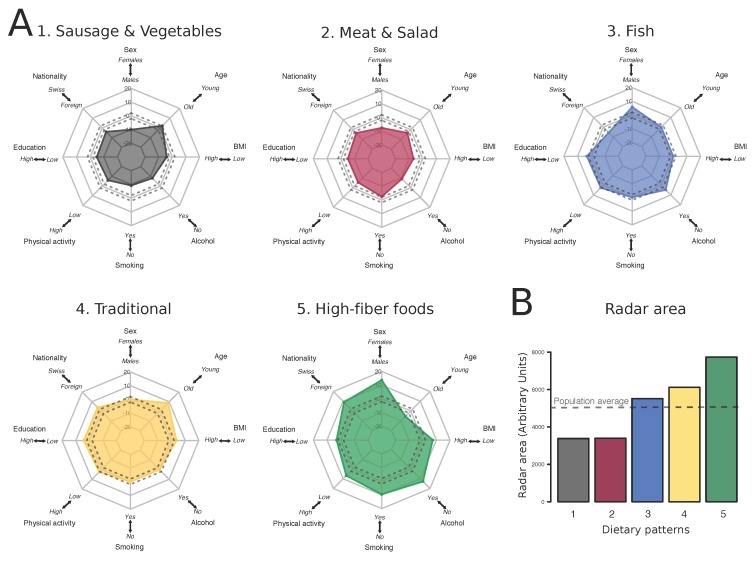
Association between dietary patterns and demographics and lifestyle behaviors (*N* = 15,936). (**A**) v-test statistics between individuals assigned to a dietary pattern and the overall population for the eight covariates included in the study. v-test indicates whether categories of participants are significantly over- (v-test > 1.96) or underrepresented (v-test < –1.96) in a dietary pattern compared to the overall study population. Grey dashed lines indicate the non-significant range (−1.96; 1.96). For representation purposes, the six categorical covariates were dichotomized (BMI: “low” < 25 kg/m^2^ vs. “high” ≥ 25 kg/m^2^; Alcohol: “no” vs. “yes” moderate or high consumption; Smoking: “no” never vs. “yes” former, light or heavy smoker; Physical activity: “low” ≤ once a week vs. “high” ≥ twice a week; Education: “low” mandatory education vs. “high” upper secondary and tertiary education; Nationality: “Swiss” vs “Foreign”); (**B**) Areas of the radar plots shown in A (arbitrary units). Horizontal dashed line indicates the radar area of the total study population (all v-tests = 0).

In the two low-variety dietary patterns (“Sausage and Vegetables” and “Meat and Salad”), foreign nationals, males and high BMI individuals as well as unhealthy behaviors (drinking, smoking, low physical activity) were overrepresented whereas higher education (Figure 2A) was underrepresented. Remarkably, the “High-fiber foods” pattern showed the exact opposite profile. Moreover, the “traditional” pattern showed an overrepresentation of Swiss nationals with low BMI, but was not associated with one sex or with healthy behaviors (Figure 2A). Finally, the “Fish” cluster showed an overrepresentation of foreign nationals, females, and of some, but not all healthy behaviors (Figure 2A). Because the plots were constructed to have mortality-reducing factors pointing outwards, radar areas visually confirmed the association between dietary patterns and such factors (Figure 2B).

### 3.4. Association between Dietary Patterns and Mortality

The “Fish” and the “Traditional” dietary patterns were inversely associated with all-cause mortality (Table 2). Sex-stratified analyses indicated that these associations were only significant in men (hazard ratio (HR) = 0.82, 95% CI (0.71–0.96); 0.81 (0.71–0.93)). Disease-specific mortality showed further sex differences. First, the “Fish” pattern was associated with a reduced cardiovascular mortality in men (HR = 0.73 (0.55–0.97)) but not in women (HR = 1.24 (0.93–1.65)). Conversely, a significant reduction of the risk of dying from cancer was only evident in women following the “High-fiber foods” pattern (HR = 0.77 (0.60–0.99)), whereas only trends were detected for men (HR = 0.92 (0.74–1.16)).

Based on these results, we estimated the PAFs (Table S3) and RAPs (Table S4) of exposure to the two low-variety dietary patterns “Sausage and Vegetables” and “Meat and Salad”. The fraction of deaths attributable to low-variety dietary patterns was 3.26% (95% CI (0.46%, 6.09%)) and exposure to a low-variety dietary pattern resulted in a 0.70 year (95% CI (0.64 year, 0.75 year)) advancement in all-cause mortality rates. Cancer mortality showed the highest PAF (5.07% (0.31%, 9.86%)) and RAP (1.60 year (1.49 year, 1.69 year)). In addition, PAFs and RAPs were systematically larger in men than women, for all-cause and disease-specific mortality.

## 4. Discussion

Using MCA and hierarchical clustering, we identified five dietary patterns in the population of Switzerland (1977–1993) and examined their associations with demographics, behaviors and mortality. In contrast to individuals consuming a low-variety dietary pattern, individuals consuming a “Fish”, “Traditional” or “High-fiber foods” pattern displayed more healthy behaviors (no smoking, no drinking, high physical activity). In addition, consumption of one of these three patterns was associated with a reduced all-cause and/or disease-specific mortality. 

In our study, we observed both often-reported and more specific types of dietary patterns. The two low-variety patterns (“Sausage and Vegetables” and “Meat and Salad”) can be compared to patterns classically labeled as “monotonous”, “conservative” or “low diversity”. Although those patterns were detected also in vegetarian groups [23], they were here characterized by the consumption of processed or unprocessed meat. Other frequently reported patterns are the “healthy”, “health conscious” or “prudent” patterns [24,25,26,27], which are best in line with our “High-fiber foods” pattern. “Fish” patterns were also reported in the literature, either as a main diet feature [28] or as feature of “healthy” patterns [29]. Interestingly, the pattern we named “Traditional” displayed features of a typical Swiss diet (i.e., high consumption of milk, dairy products, and chocolate [3]), but was also characterized by certain elements found in “Western” patterns [24,25,27] (i.e., high consumption of energy-dense products and processed meat). Being essentially found in young Swiss nationals, this pattern may therefore indicate a hybrid diet that integrates both the classic Swiss culinary traditions with more modern “Western” consumption habits. 

Furthermore, our data confirm previous results indicating that “healthy” patterns are more frequently followed by women with low BMI, high education and high physical activity level [23,28], whereas “monotonous” dietary patterns show the opposite profile. Similarly, our results for the “Fish” pattern partially confirm previous evidence showing that fish consumers have on average a healthier lifestyle than non-consumers [30,31]. More generally, our results highlight that dietary patterns remarkably cluster with other protective or risk factors and confirm that health behaviors are interrelated [32].

Consistent with meta-analysis [33] and large European cohort studies [34,35], we found significant associations between dietary patterns that are similar to a prudent diet (“High-fiber foods” pattern) or a Mediterranean diet (“Fish” pattern) and a reduced all-cause, cardiovascular or cancer mortality. Interestingly, all patterns characterized by a high dietary variety were associated with a reduced mortality. This result corroborates the concept that dietary variety can be considered a hallmark of healthy diets: a priori-defined scores, such as the Healthy Eating Index, also incorporate dietary variety as a component of diet evaluation [19] and dietary variety was repeatedly linked with nutrient adequacy [36,37,38]. In addition, although the nutrient composition of the dietary patterns could not be calculated in this study, it appears plausible that certain nutrients may have mediated the effects of dietary patterns on mortality. For example, it is likely that participants following the “Fish” pattern had higher intake of omega-3 fatty acids, which were linked with reduced cardiovascular [39] and cancer [40] risks. Similarly, in the “High-fiber foods” pattern, a high intake of dietary fibers plausibly mediates the association between this dietary pattern and a reduced cancer mortality [41].

Furthermore, sex differences were evident in the association between dietary patterns and mortality. Recently, a study in Japanese adults also indicated that the association between diet and all-cause mortality, as well as cardiovascular mortality, was more evident in men [6]. Interestingly, the “High-fiber foods” pattern showed reduced cancer mortality in women only. Possible explanations include the fact that men and women are susceptible to different cancer types. Therefore, it is possible that our findings reflect the different susceptibility of cancer types to nutritional influences (see continuous update matrix of the World Cancer Research Fund International for summary [41]). On another note, effects of dietary patterns on cancer mortality in men were strongly diminished after adjustment for other lifestyle factors. Thus, it is possible that these other factors contribute more to cancer mortality in men than women, finally reducing nutritional influences [42]. Overall, our data further suggests that the association between diets and mortality should account for potential interaction between sex and other disease risk factors. 

Our study has strengths and limitations. First, our study includes a large and nationwide representative sample. Moreover, the mortality follow-up obtained by the SNC is longer than in most studies and allows for a long-term monitoring of dietary influences and high regression power. Only a limited number of covariates, however, were available for this study: therefore, the possibility of residual confounding cannot be excluded. Our dietary patterns are based on a single 24-h recall, which is adequate for surveying intake in a large group but does not necessarily reflect an individual’s habitual diet. In addition, dietary assessment was done once at baseline and it is possible that dietary habits changed during follow-up. Therefore, conclusions on the association between diet and mortality should be interpreted bearing these limitations in mind. In addition, diets were recorded using a 24-h recall checklist (i.e., as binary intakes) rather than a classic 24-h recall (i.e., continuous intakes in grams). Therefore, we used MCA (applicable on categorical variables) rather than the most commonly used principal component analysis to study dietary patterns (see [4,43] for review). When compared, however, both methods led to meaningful and similar dietary patterns [44], suggesting that binary intakes can be interpreted as general food preferences [44]. Nevertheless, a qualitative dietary assessment does neither allow for inferring nutrient intake nor for assessing the nutrients potentially mediating the association between dietary patterns and disease. Also, the dietary assessment conducted in NRP1A and MONICA studies grouped foods in broad categories, which prevented us from analyzing food groups that may have been more relevant from a diet-disease association perspective (i.e., meat types). Like a few others [28,45,46], we used a two-step strategy in the identification of dietary patterns, following up MCA with hierarchical clustering. This strategy allows first for removal of the non-interpretable variation (“noise”) in diets and second for the establishment of discrete meaningful dietary patterns on the remaining interpretable variation [28]. Therefore, supplementary dietary patterns might have been eliminated during the pre-processing with MCA, but these patterns would not be clearly supported by the data and would be difficult to interpret. Finally, we acknowledge that both principal components and clustering methods are partially based on arbitrary analytic decisions (number of dimensions or clusters to retain, for example [18]) and may show limited stability [47,48] or reproducibility [29,49,50,51]

## 5. Conclusions

In conclusion, our study leverages multiple exploratory methods to study dietary patterns and their associations with demographics, healthy behaviors and mortality. Dietary patterns and other healthy behaviors were interrelated, suggesting that integrative public health measures may synergistically reduce disease risk. Our data also suggest that, beyond dietary patterns, broadening the dietary choices, within the framework of personal food preferences, could be one simple way to improve the quality of the diets and reduce the burden of chronic diseases.

## Figures and Tables

**Table 1:** Characteristics of the study participants by sex ^1^.

	Overall	Females	Males	Missing
Total	15,936 (100)	8143 (51.1)	7793 (48.9)	
Number of deaths	4630 (29.1)	2077 (25.5)	2553 (32.8)	
Age, year	45.0 ± 13.5	45.2 ± 13.8	44.8 ± 13.1	
Survival time, year	25.5 ± 9.1	26.3 ± 8.6	24.6 ± 9.4	
BMI				15 (0.1)
<25 kg/m^2^	8845 (55.5)	5317 (65.3)	3528 (45.3)	
25–30 kg/m^2^	5503 (34.5)	2069 (25.4)	3434 (44.1)	
≤30 kg/m^2^	1573 (9.9)	752 (9.2)	821 (10.5)	
Nationality				0 (0)
Swiss	12,949 (81.3)	6829 (83.9)	6120 (78.5)	
Foreign	2987 (18.7)	1314 (16.1)	1673 (21.5)	
Education				20 (0.1)
Mandatory	5504 (34.5)	3339 (41.0)	2165 (27.8)	
Upper secondary	7567 (47.5)	3649 (44.8)	3918 (50.3)	
Tertiary	2845 (17.9)	1143 (14.0)	1702 (21.8)	
Physical activity				253 (1.6)
<1×/week	8722 (54.7)	4534 (55.7)	4188 (53.7)	
1×/week	3497 (21.9)	1891 (23.2)	1606 (20.6)	
>1×/week	3464 (21.7)	1568 (19.3)	1896 (24.3)	
Smoking				25 (0.2)
Never	7533 (47.3)	4945 (60.7)	2588 (33.2)	
Former	2699 (16.9)	822 (10.1)	1877 (24.1)	
Light	3232 (20.3)	1601 (19.7)	1631 (20.9)	
Heavy	2447 (15.4)	758 (9.3)	1689 (21.7)	
Alcohol				93 (0.6)
No	7087 (44.5)	4896 (60.1)	2191 (28.1)	
Moderate	5971 (37.5)	2571 (31.6)	3400 (43.6)	
High	2785 (17.5)	622 (7.6)	2163 (27.8)	

^1^ Values are *N* (%) or means ± standard deviations; 1×: once a week.

**Table 2:** Association between dietary patterns and all-cause or disease-specific mortality ^1^.

	Overall		Women		Men	
	Basic	Multivariable	Basic	Multivariable	Basic	Multivariable
ALL CAUSE						
Number of deaths	*n* = 4264		*n* = 1947		*n* = 2317	
“Sausage and Vegetables”	1	1	1	1	1	1
“Meat and Salad”	0.92 (0.84–1.00)	0.94 (0.86–1.03)	0.90 (0.77–1.04)	0.93 (0.80–1.08)	0.93 (0.82–1.04)	0.95 (0.85–1.07)
“Fish”	**0.79 (0.71–0.88)**	**0.87 (0.78–0.97)**	0.93 (0.79–1.09)	0.98 (0.83–1.15)	**0.77 (0.67–0.90)**	**0.82 (0.71–0.96)**
“Traditional”	**0.81 (0.73–0.90)**	**0.89 (0.80–0.98)**	0.84 (0.80–1.10)	1.02(0.87–1.19)	**0.75 (0.65–0.85)**	**0.81 (0.71–0.93)**
“High-fiber foods”	**0.76 (0.69–0.83)**	0.92 (0.84–1.02)	**0.82 (0.72–0.95)**	0.91 (0.79–1.05)	**0.84 (0.74–0.95)**	0.94 (0.83–1.08)
CARDIOVASCULAR DISEASE						
Number of deaths	*n* = 1432		*n* = 662		*n* = 770	
“Sausage and Vegetables”	1	1	1	1	1	1
“Meat and Salad”	1.01 (0.86–1.19)	1.02 (0.87–1.20)	1.05 (0.81–1.37)	1.07 (0.82–1.39)	0.97 (0.78–1.19)	0.98 (0.50–1.21)
“Fish”	0.85 (0.70–1.03)	0.91 (0.75–1.11)	1.22 (0.92–1.63)	1.24 (0.93–1.65)	**0.69 (0.52–0.91)**	**0.73 (0.55–0.97)**
“Traditional”	**0.83 (0.69–1.00)**	0.87 (0.73–1.04)	1.00 (0.75–1.32)	1.06 (0.79–1.41)	**0.73 (0.57–0.93)**	**0.76 (0.59–0.98)**
“High-fiber foods”	**0.84 (0.71–0.99)**	0.99 (0.85–1.18)	0.92 (0.72–1.18)	0.98 (0.76–1.26)	0.94 (0.75–1.17)	1.02 (0.81–1.28)
CANCER						
Number of deaths	*n* = 1460		*n* = 634		*n* = 826	
“Sausage and Vegetables”	1	1	1	1	1	1
“Meat and Salad”	0.91 (0.78–1.06)	0.95 (0.82–1.11)	0.83 (0.65–1.07)	0.88 (0.68–1.12)	0.96 (0.80–1.17)	0.95 (0.82–1.20)
“Fish”	**0.72 (0.60–0.86)**	**0.82 (0.68–0.99)**	**0.71 (0.54–0.94)**	0.77 (0.58–1.02)	0.80 (0.63–1.03)	0.87 (0.68–1.12)
“Traditional”	**0.83 (0.70–0.98)**	0.93 (0.79–1.10)	0.95 (0.74–1.23)	1.04 (0.81–1.35)	**0.77 (0.62–0.97)**	0.86 (0.69–1.08)
“High-fiber foods”	**0.68 (0.58–0.80)**	**0.85 (0.72–1.00)**	**0.69 (0.54–0.88)**	**0.77 (0.60–0.99)**	**0.79 (0.63–0.98)**	0.92 (0.74–1.16)
OTHER CAUSES						
Number of deaths	*n* = 1372		*n* = 651		*n* = 721	
“Sausage and Vegetables”	1	1	1	1	1	1
“Meat and Salad”	**0.83 (0.71–0.98)**	0.86 (0.73–1.01)	0.83 (0.64–1.09)	0.86 (0.66–1.13)	0.96 (0.80–1.17)	0.99 (0.82–1.20)
“Fish”	**0.81 (0.67–0.97)**	0.89 (0.73–1.08)	0.94 (0.71–1.26)	0.99 (0.74–1.32)	0.80 (0.63–1.08)	0.87 (0.68–1.12)
“Traditional”	**0.79 (0.66–0.94)**	0.86 (0.72–1.03)	0.87 (0.67–1.15)	0.95 (0.72–1.25)	**0.77 (0.62–0.97)**	0.86 (0.69–1.08)
“High-fiber foods”	**0.77 (0.65–0.90)**	0.93 (0.79–1.10)	0.88 (0.69–1.12)	0.99 (0.77–1.26)	**0.79 (0.63–0.98)**	0.92 (0.74–1.16)

^1^ Values are hazard ratios (and 95% confidence intervals). An additional category was created for missing information on covariates so that the whole sample was available for Cox regression. Hazard ratios significantly different to 1 appear in bold. Cox regression models were adjusted for potential confounders in a sequential manner; basic model: study (MONICA wave 1–3; NRP1A); multivariable model: study, BMI, nationality, education, smoking status, alcohol consumption and physical activity.

## Supplementary Materials

The following are available online at http://www.mdpi.com/2072-6643/10/3/313/s1, Figure S1: A. Scree plot of the first 12 principal components of the multiple correspondence analysis. B. Percentage of variance (i.e., inertia) and cumulative percentage of variance explained by the first 12 principal components of the multiple correspondence analysis. C. Projection of the individuals (i.e., study participants) in the first 2 principal components and D. in the third and fourth principal components of the multiple correspondence analysis. E. Projection of the food group yes/no categories in the first 2 principal components and F. in the third and fourth principal components of the multiple correspondence analysis. Figure S2: A. Hierarchical tree obtained by hierarchical clustering on the first 4 principal components of the multiple correspondence analysis. B. Changes in between-inertia between partitions containing *n* or *n* + 1 clusters. C. Projection of the clustered individuals (i.e., study participants) in the first 2 principal components and D. in the third and fourth principal components of the multiple correspondence analysis. E. Number (*N*) and percentage (%) of study participants assigned to the five dietary patterns. Table S1: Consumption of the 12 food groups among the study participants (number and percentage). Table S2: Factor loadings of the first four dimensions of the multiple correspondence analysis. Table S3: Population Attributable Fractions: fractions of all-cause and disease-specific mortality attributable to low-variety dietary patterns, Table S4: Rate of Advancement Periods: years of advancement in mortality rates due to low-variety dietary patterns.
