# Supplementary material for: Dietary Patterns Are Associated with Cardiovascular and Cancer Mortality among Swiss Adults in a Census-Linked Cohort
Source: Nutrients. 2018 Mar 7;10(3):313. doi: 10.3390/nu10030313 (PMC5872731; doi:10.3390/nu10030313)
Supplement: Supplementary file 1 [file nutrients-10-00313-s001.pdf]

Figure S1

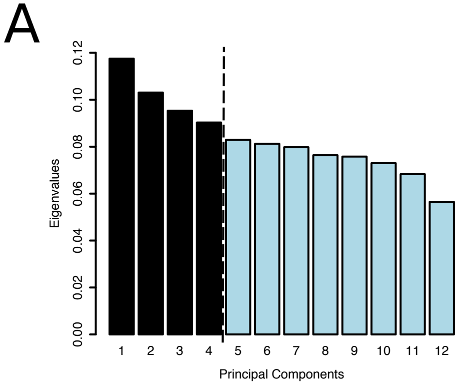

B

	percentage of variance	cumulative percentage of variance
dim 1	11.75	11.75
dim 2	10.31	22.06
dim 3	9.53	31.59
dim 4	9.03	40.62
dim 5	8.29	48.91
dim 6	8.13	57.04
dim 7	7.98	65.01
dim 8	7.64	72.65
dim 9	7.58	80.23
dim 10	7.30	87.52
dim 11	6.83	94.35
dim 12	5.65	100.00

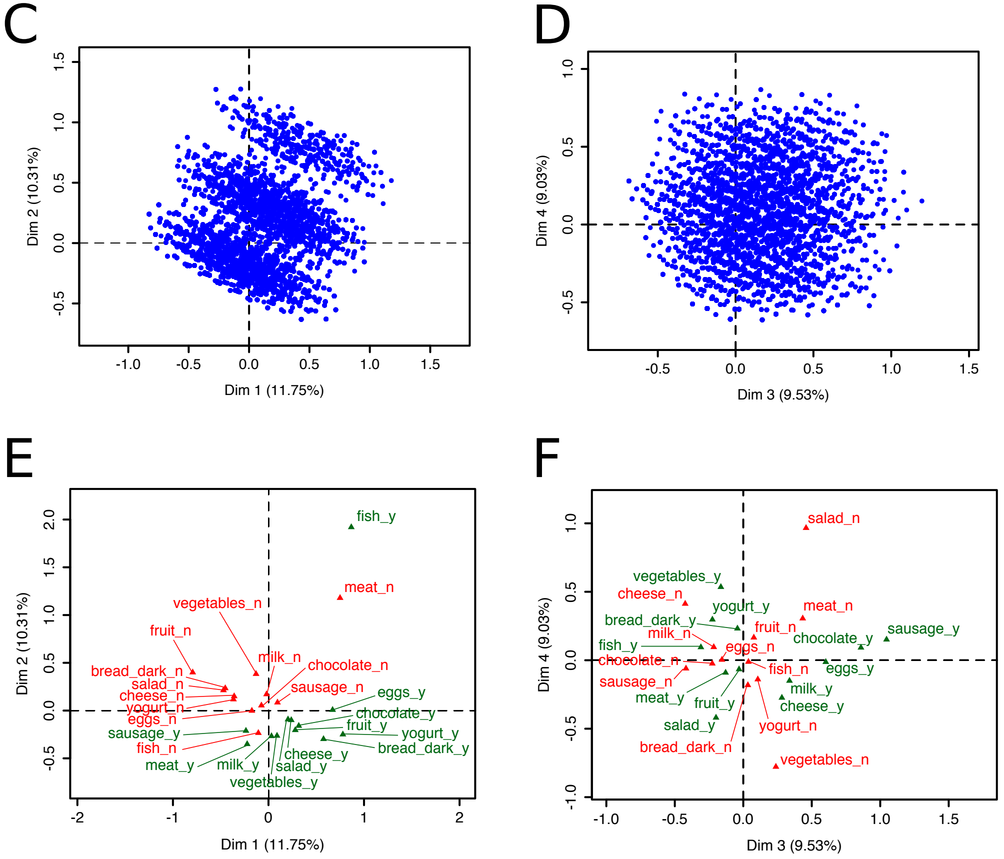

Figure S2

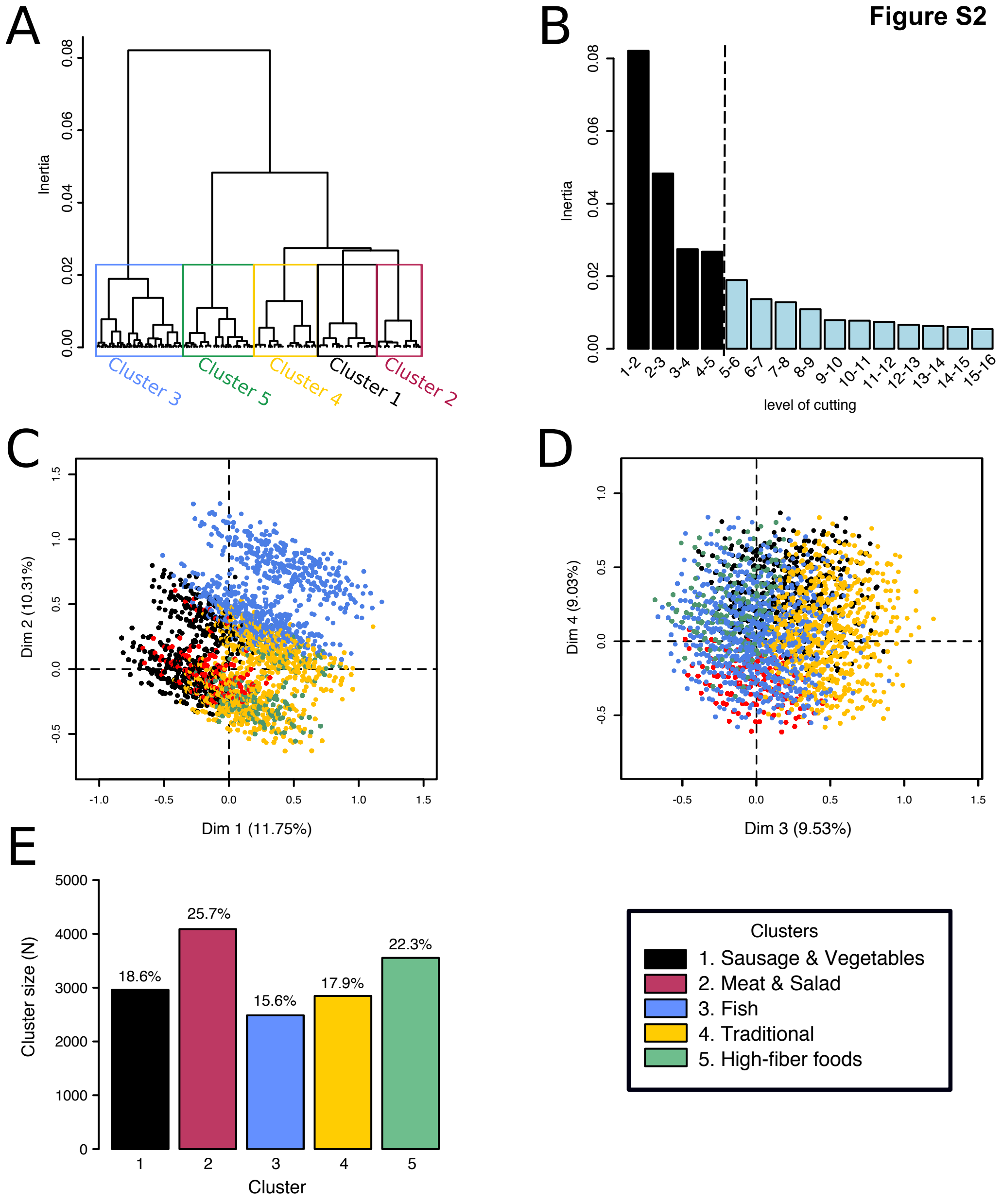
A
Inertia
0.00
0.02
0.04
0.06
0.08
Cluster 3
Cluster 5
Cluster 4
Cluster 1
Cluster 2
B
Inertia
0.00
0.02
0.04
0.06
0.08
1-2
2-3
3-4
4-5
5-6
6-7
7-8
8-9
9-10
10-11
11-12
12-13
13-14
14-15
15-16
level of cutting
C
Dim 2 (10.31%)
-0.5
0.0
0.5
1.0
1.5
-1.0
-0.5
0.0
0.5
1.0
1.5
Dim 1 (11.75%)
D
Dim 4 (9.03%)
-0.5
0.0
0.5
1.0
-0.5
0.0
0.5
1.0
1.5
Dim 3 (9.53%)
E
Cluster size (N)
0
1000
2000
3000
4000
5000
18.6%
25.7%
15.6%
17.9%
22.3%
1
2
3
4
5
Cluster
Clusters
1. Sausage & Vegetables
2. Meat & Salad
3. Fish
4. Traditional
5. High-fiber foods

Table S1: Consumption of the 12 food groups among the study participants (number and percentage)

	Overall		Females		Males	
	N	%	N	%	N	%
meat	12282	77.1	5990	73.6	6292	80.7
fruit	11384	71.4	6381	78.4	5003	64.2
salad	11111	69.7	5753	70.6	5358	68.8
cheese	9574	60.1	4713	57.9	4861	62.4
vegetables	9445	59.3	4979	61.1	4466	57.3
bread_dark	7009	44.0	3828	47.0	3181	40.8
milk	6213	39.0	3028	37.2	3185	40.9
yogurt	5099	32.0	3079	37.8	2020	25.9
sausage	4552	28.6	1975	24.3	2577	33.1
chocolate	3323	20.9	1772	21.8	1551	19.9
eggs	3276	20.6	1781	21.9	1495	19.2
fish	1734	10.9	868	10.7	866	11.1

Table S2: Factor loadings of the first four dimensions of the multiple correspondence analysis[1]

Dim. 1 "low vs. high variability"	yes	no
fish	2.53	-0.31
fruit	0.93	-2.31
yogurt	2.27	-1.07
meat	-0.65	2.19
eggs	1.96	-0.51
bread_dark	1.68	-1.32
salad	0.60	-1.37
cheese	0.70	-1.05
chocolate	0.82	
sausage	-0.69	
vegetables		-0.38

Dim. 2 "meat vs. fish"	yes	no
fish	5.98	-0.73
meat	-1.09	3.67
fruit	-0.50	1.24
vegetables	-0.82	1.19
bread_dark	-0.93	0.73
milk	-0.83	0.53
yogurt	-0.78	0.37
salad		0.66
sausage	-0.66	
chocolate	-0.64	
cheese	-0.31	0.47

Dim. 3 "high energy vs. high fiber"	yes	no
sausage	3.39	-1.35
chocolate	2.78	-0.73
eggs	1.95	-0.50
salad	-0.64	1.48
meat	-0.42	1.41
cheese	0.91	-1.37
milk	1.09	-0.70
fish	-1.00	
vegetables	-0.53	0.77
yogurt	-0.73	0.34

Dim. 4 "salad vs. vegetables"	yes	no
salad	-1.40	3.21
vegetables	1.78	-2.59
cheese	-0.91	1.37
meat	-0.30	1.02
yogurt	0.99	-0.47
bread_dark	0.77	-0.61
fruit		0.55
sausage	0.50	
milk	-0.50	0.32
fish	0.32	
chocolate	0.31	

[1]Factor loadings <|0.3| are not listed; food groups were ranked by the maximum of their „yes“ or „no“ loadings in each dimension; when none of the 2 factor loadings of a food group were <|0.3|, the food group was removed for clarity).

Table S3: Population Attributable Fractions: fractions of all-cause and disease-specific mortality attributable to low-variety dietary patterns.[1]

	Overall		Women		Men	
	Basic	Multivariable	Basic	Multivariable	Basic	Multivariable
ALL CAUSE	**8.76%**	**3.26%**	2.69%	0.17%	**9.67%**	**5.97%**
	**[6.00%, 11.52%]**	**[0.46% ,6.09%]**	[-0.97%, 6.42%]	[-3.51%, 3.92%]	**[5.47%, 13.84%]**	**[1.66%, 10.27%]**
CARDIOVASCULAR DISEASE	**8.14%**	3.52%	0.91%	-0.75%	**10.07%**	7.57%
	**[3.32%, 12.97%]**	[-1.39%, 8.47%]	[-5.36%, 7.40%]	[-7.08%, 5.84%]	**[2.63%, 17.40%]**	[-0.10%, 15.15%]
CANCER	**11.19%**	**5.07%**	**6.49%**	3.72%	**10.95%**	5.83%
	**[6.51%, 15.89%]**	**[0.31%, 9.86%]**	**[0.15%, 12.99%]**	[-2.65%, 10.29%]	**[3.95%, 17.84%]**	[-1.35%, 12.95%]
OTHER CAUSES	**6.42%**	1.01%	0.49%	-2.34%	7.10%	3.47%
	**[1.55%, 11.32%]**	[-3.93%, 6.01%]	[-5.78%, 7.00%]	[-8.62%, 4.22%]	[-0.45%, 14.57%]	[-4.26%, 11.16%]

[1] Values are PAFs [95% confidence intervals]. „Basic“ and „Multivariable“ indicate PAFs calculated on the basis of the proportional hazards Cox regression models adjusted for study (“Basic”) or for study, BMI, nationality, education, smoking status, alcohol consumption and physical activity (”Multivariable”).

Table S4: Rate of Advancement Periods: years of advancement in mortality rates due to low-variety dietary patterns[1].

	Overall		Women		Men	
	Basic	Multivariable	Basic	Multivariable	Basic	Multivariable
ALL CAUSE	**1.89 y**	**0.70 y**	**0.58 y**	0.01 y	**2.02 y**	**1.23 y**
	**[1.83, 1.94]**	**[0.64, 0.75]**	**[0.50, 0.67]**	[-0.07, 0.10]	**[1.94, 2.09]**	**[1.15, 1.31]**
CARDIOVASCULAR DISEASE	**1.29 y**	**0.55 y**	**0.20 y**	-0.07 y	**1.51 y**	**1.02 y**
	**[1.20, 1.39]**	**[0.45, 0.64]**	**[0.06, 0.33]**	[-0.21, 0.07]	**[1.39, 1.64]**	**[0.89, 1.15]**
CANCER	**3.68 y**	**1.60 y**	**2.57 y**	**1.40 y**	**3.04 y**	**1.66 y**
	**[3.58, 3.77]**	**[1.49, 1.69]**	**[2.42, 2.72]**	**[1.25, 1.55]**	**[2.91, 3.17]**	**[1.53, 1.79]**
OTHER CAUSES	**1.24 y**	**0.16 y**	**-0.15 y**	**-0.72 y**	**1.73 y**	**1.07 y**
	**[1.14, 1.34]**	**[0.06, 0.25]**	**[-0.30, -0.01]**	**[-0.86, -0.57]**	**[1.60, 1.87]**	**[0.93, 1.21]**

[1] Values are RAPs in years [95% confidence intervals]. „Basic“ and „Multivariable“ indicate RAPs calculated on the basis of the proportional hazards Cox regression models adjusted for study (“Basic”) or for study, BMI, nationality, education, smoking status, alcohol consumption and physical activity (”Multivariable”)
